# Porcine Enteric Coronavirus PEDV Induces the ROS-ATM and Caspase7-CAD-γH2AX Signaling Pathways to Foster Its Replication

**DOI:** 10.3390/v14081782

**Published:** 2022-08-15

**Authors:** Xin Ming, Huan Chen, Ying Yang, Pu Zhao, Liumei Sun, Caisheng Zhang, Hyun-Jin Shin, Jeong-Soo Lee, Yong-Sam Jung, Yingjuan Qian

**Affiliations:** 1 MOE Joint International Research Laboratory of Animal Health and Food Safety, Jiangsu Foreign Expert Workshop, College of Veterinary Medicine, Nanjing Agricultural University, Nanjing 210095, China; 2 Jiangsu Key Laboratory of Sericultural Biology and Biotechnology, School of Biotechnology, Jiangsu University of Science and Technology, Zhenjiang 212100, China; 3 College of Veterinary Medicine, Chungnam National University, Daejeon 305-764, Korea; 4 Department of Electrical Engineering, Pohang University of Science and Technology (POSTECH), Pohang 37673, Korea; 5 Jiangsu Key Laboratory for High-Tech Research and Development of Veterinary Biopharmaceuticals, Jiangsu Agri-Animal Husbandry Vocational College, Taizhou 225300, China

**Keywords:** PEDV, ATM, γH2AX, caspase, DNA damage response

## Abstract

DNA damage response (DDR) is an evolutionarily conserved mechanism by which eukaryotic cells sense DNA lesions caused by intrinsic and extrinsic stimuli, including virus infection. Although interactions between DNA viruses and DDR have been extensively studied, how RNA viruses, especially coronaviruses, regulate DDR remains unknown. A previous study showed that the porcine epidemic diarrhea virus (PEDV), a member of the genus *Alphacoronavirus* in the *Coronaviridae* family, induces DDR in infected cells. However, the underlying mechanism was unclear. This study showed that PEDV activates the ATM-Chk2 signaling, while inhibition of ATM or Chk2 dampens the early stage of PEDV infection. Additionally, we found that PEDV-activated ATM signaling correlates with intracellular ROS production. Interestingly, we showed that, unlike the typical γH2AX foci, PEDV infection leads to a unique γH2AX staining pattern, including phase I (nuclear ring staining), II (pan-nuclear staining), and III (co-staining with apoptotic bodies), which highly resembles the apoptosis process. Furthermore, we demonstrated that PEDV-induced H2AX phosphorylation depends on the activation of caspase-7 and caspase-activated DNAse (CAD), but not ATM-Chk2. Finally, we showed that the knockdown of H2AX attenuates PEDV replication. Taken together, we conclude that PEDV induces DDR through the ROS-ATM and caspase7-CAD-γH2AX signaling pathways to foster its early replication.

## 1. Introduction

Viruses have evolved sophisticated tactics to optimize the intracellular environment by hijacking cellular signaling pathways for their replication. The DNA damage response (DDR) is a complex three-tiered signaling cascade that is mediated by ataxia telangiectasia mutated (ATM), ATM and Rad3 related (ATR), and DNA-dependent protein kinase (DNA-PK), leading to DNA repair, cell cycle arrest, and apoptosis according to the extent of damage [1,2,3,4]. As a causative factor in oncogenesis and chronic inflammation, the interplay between DDR and oncogenic viruses has been extensively studied. However, many non-oncogenic RNA viruses can also induce DDR [5]. For example, hepatitis C virus (HCV) infection stimulates the production of Nitric oxide (NO) and Reactive oxygen species (ROS) and subsequently induces DNA damage and genetic abnormalities [6,7]. Infectious bronchitis virus (IBV), a member of the *Coronaviridae* family, activates the replicative stress and ATR pathway through the interaction between the nsp13 and DNA polymerase δ, leading to the S-phase arrest, which benefits viral replication [8]. A recent study showed that severe acute respiratory syndrome coronavirus 2 (SARS-CoV-2) infection also activates the ATR-Chk1 pathway and induces H2AX phosphorylation [9].

Porcine epidemic diarrhea (PED) is an acute and highly contagious enteric disease characterized by severe watery diarrhea, dehydration, and anorexia in piglets [10]. The causative agent is the porcine epidemic diarrhea virus (PEDV), an enveloped, single-stranded positive-sense RNA virus, which belongs to the family *Coronaviridae*, genus *Alphacoronavirus* [11]. The PEDV genome is approximately 28 kb in size and encodes replicase polyprotein (pp) la and pp1ab, which are subsequently cleaved into sixteen non-structural proteins (NSPs), four structural proteins, spike (S), membrane (M), envelope (E), nucleocapsid (N), and one accessory protein ORF3 [12]. In 2013, PED emerged in the United States and rapidly spread across North America and Asia, causing a devastating impact on the global swine industry [13,14,15,16]. To unravel its pathogenesis, many researchers endeavor to explore the interaction between PEDV and many important host signaling pathways including autophage, innate immune response and apoptosis [17,18,19,20,21,22]. Studies showed that the pathological changes of PEDV infection in vivo were related to the activation of the cellular apoptotic pathway [23,24,25]. In addition, PEDV infection induces the phosphorylation of Chk2 and H2AX, resulting in cell cycle arrest in the G1/G0 phase. However, the molecular mechanism of PEDV regulating the cellular DDR and the significance of DDR on PEDV replication remain unclear.

In this study, we further investigated the interaction between DDRs and PEDV infection. We showed that PEDV infection strongly activates the ATM-Chk2 signaling pathway in both Vero-E6 and Marc145 cells. Interestingly, we observed a unique γH2AX staining pattern that differs from the typical DDR foci. In addition, we found that the activation of ATM signaling is associated with the elevated level of cellular ROS, whereas the phosphorylation of H2AX is independent of ATM activity, but associated with caspase and CAD activation. Moreover, inhibition of ATM signaling by a specific inhibitor or siRNA interference largely attenuates PEDV early replication. Consistently, we demonstrated that H2AX plays a positive role in PEDV replication. Taken together, these findings suggest that PEDV infection regulates ATM-Chk2 signaling and H2AX phosphorylation through two independent mechanisms to promote PEDV replication.

## 2. Materials and Methods

### 2.1. Cells and Viruses

Vero-E6 and Marc145 cells were cultured in Dulbecco’s modified Eagle’s medium (DMEM, Gibco) supplemented with 10% fetal bovine serum (Pan-Biotech, Inc. Bavaria, Aidenbach, Germany) at 37 °C with 5% CO_2_. Two PEDV strains, CV777, a vaccine strain, and HLJBY (KP403802.1), a virulence-attenuated strain, were used in this study.

### 2.2. Plasmids and Reagents

*ICAD/DFFA/DFF45* (NM_004401.3) was amplified from cDNA prepared with MCF7 cells, and ICAD mutants were amplified from ICAD construct with primers listed in Table 1. PCR fragments were cloned into pcDNA3-Flag using ClonExpress Ultra One Step Cloning Kit (Vazyme Biotech, Inc. Nanjing, China). Hydroxyurea (HU), Apocynin (APO), Acetylcysteine (NAC), Diphenyleneiodonium chloride (DPI), and caspase 3/7 inhibitors Ac-DEVD-CHO, ATM inhibitor KU55933, and ATR inhibitor VE-821 were purchased from Selleck Chemicals Inc. (Houston, TX, USA). Caspase pan-inhibitor Z-VAD-FMK and caspase 8 inhibitor Z-IETD-FMK were purchased from APExBIO Technology Inc. (Houston, TX, USA). 2′,7′-Dichlorodihydrofluorescein diacetate (DCFH-DA) were purchased from Merck Inc. (Darmstadt, Germany).

### 2.3. Western-Blot Analysis

Whole-cell extracts were prepared with 2 × SDS sample buffer (0.1 M Tris HCI, pH 6.8, 20% glycerol, 4% SDS, 10% β-Mercaptoethanol, 2% Bromophenol blue) and boiled for 10 min at 98 °C. Rabbit anti-γH2AX (S139) (9718), -H2AX (2595), -pATM (S1981) (13050), -pChk2 (T68) (2661), -Chk2 (2662), -pChk1 (S317), -PARP (9542), -caspase 7 (9492), -cleaved caspase 3 (D175) (9661), mouse anti-DNA-PK (3H6), and -caspase 8 (9746) were purchased from Cell Signaling Technology (Danvers, MA, USA). Mouse anti-ATM (1A1), -Chk1 (G-4), -CAD (F-11), goat anti-ATR (N-19), and rabbit anti-caspase 3 (H-277) were purchased from Santa Cruz Biotechnology (Dallas, TX, USA). Rabbit anti-pATR (T1989) was purchased from GeneTex (Irvine, CA, USA). Rabbit anti-pDNA-PK (S2056) was purchased from Abcam (Cambridge, UK). Mouse anti-Flag (M-2) was purchased from Sigma-Aldrich (St. Louis, MO, USA). Rabbit anti-actin (20536) was purchased from Proteintech Group (Rosemont, IL, USA). Rabbit anti-PEDV N was generated previously in our lab [26]. The horseradish peroxidase (HRP)-conjugated goat anti-rabbit IgG antibody and HRP-conjugated goat anti-mouse IgG antibody were purchased from MiliporeSigma (Merck Inc., Darmstadt, Germany).

### 2.4. RNA Isolation and Reverse Transcription (RT)-Polymerase Chain Reaction (PCR)

Total RNA was extracted using TRIzol reagent (MiliporeSigma, Merck Inc., Darmstadt, Germany), and the cDNA was obtained using the HiScript II Q RT SuperMix for qPCR (+gDNA wiper) following the manufacturer’s instructions (Vazyme Biotech, Inc. Nanjing, China). The PCR was carried out with primers targeting *ORF3* and *GAPDH* listed in Table 1 using 2 × Taq Master Mix (Vazyme Biotech, Inc., Nanjing, China) under the following conditions: denaturation at 94 °C for 5 min, followed by 25 cycles of 94 °C for 30 s, annealing at 55 °C for 30 s, extension at 72 °C for 30 s, and a final extension at 72 °C for 10 min.

### 2.5. Indirect Immunofluorescence Assay (IFA)

IFA was performed as previously described [26]. Briefly, Vero-E6 cells were seeded at 8 × 10^5^ on glass coverslips in a six-well plate, followed by mock-treated or infected with PEDV (MOI = 1) for indicated times. Cells were fixed and stained with their respective antibodies and visualized by the confocal microscope (Nikon Inc., Tokyo, Japan).

### 2.6. Transient Transfection and siRNA Knockdown

The experiments were performed as previously described [27]. siRNAs against *caspase-3*, *caspase-7*, *ATM*, *Chk2*, *H2AX*, and scramble siRNA are listed in Table 2 (Biotend Inc., Shanghai, China).

### 2.7. ROS Analysis

Vero-E6 or Marc145 cells were infected with PEDV at different times and harvested at the same time. Then, cells were incubated with 10 μM 2′,7′-Dichlorodihydrofluorescein diacetate (DCFH-DA) at 37 °C for 30 min in dark. After rinsing three times with PBS, cells were trypsinized and resuspended in PBS and then analyzed by FACS Calibur flow cytometer (BD Biosciences Inc., San Jose, CA, USA).

### 2.8. Plaque Formation Assay

Vero-E6 cells were seeded in six-well plates at 80% confluence 18 h before infection. The virus was diluted in DMEM through serial ten-fold dilution and incubated with cells at 37 °C for 1 h. Cells were washed with PBS and overlaid with 1% low-melting-point agarose (Lonza Inc., Basel, Switzerland) in the DMEM medium with 2% FBS and incubated at 37 °C for 2–3 days until the plaques formed and finally stained with 0.5% crystal violet.

### 2.9. Cell Viability Analysis

The cytotoxicity of DNA damage inhibitors was measured by CCK-8 assay following the manufacturer’s instructions (APExBIO, Houston, TX, USA). Briefly, Vero-E6 cells were seeded in the 96-well plate at 5000 per well for 12 h and then treated with an increased dose of inhibitors for 24 h, followed by incubated with CCK-8 reagent (10 μL/well) at 37 °C for 4 h. Finally, the absorbance at 450 nm was measured in an enzyme-linked immunosorbent assay reader.

### 2.10. Statistical Analysis

The data were obtained from three replicates and presented as means ± standard deviations (SD). Statistical significance between different groups was determined using the Student’s *t*-test in GraphPad Prism 7.0 software (San Diego, CA, USA) (* *p* < 0.05; ** *p* < 0.01; *** *p* < 0.001).

## 3. Results

### 3.1. PEDV Infection Activates the ATM-Chk2 Signaling Pathway

**Figure 1:**
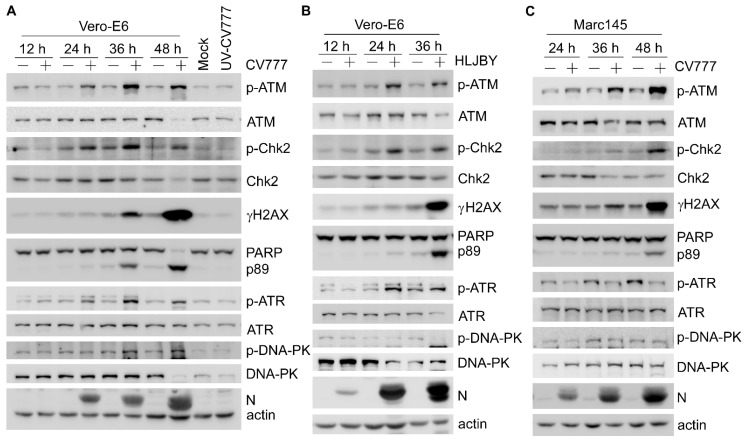
PEDV infection activates ATM-dependent DNA damage response. (**A**) Western blots were prepared with extracts from Vero-E6 cells uninfected or infected with CV777 (0.1 MOI) for 12, 24, 36, and 48 h. The levels of p-ATM (S1981), total ATM, p-Chk2(T68), total Chk2, p-ATR(T1989), total ATR, p-DNA-PK(S2056), total DNA-PK, γH2AX, PEDV N, and actin were determined using their respective antibodies. Vero-E6 cells infected with UV-inactivated CV777 (0.1 MOI) for 48 h were used as the negative control. (**B**) Western blots were prepared with extracts from Vero-E6 cells uninfected or infected with HLJBY (0.1 MOI) for 12, 24, and 36 h. (**C**) Western blots were prepared with extracts from Marc145 cells uninfected or infected with CV777 (0.1 MOI) for 24, 36, and 48 h at 0.1 MOI.

To determine whether PEDV infection regulates host DNA damage response (DDR), we first examined the activation of three apical kinases ATM, ATR, and DNA-PK, and their downstream effectors in Vero-E6 cells infected with CV777 or HLJBY. We found that both PEDV strains dramatically induced ATM phosphorylation on Ser1981, ATR phosphorylation on Thr1989, ATM substrate Chk2 phosphorylation on Thr68, and H2AX phosphorylation on Ser139 (γH2AX) at the late infection stage (Figure 1A,B), consistent with the previous observation in Vero cells upon infection with an epidemic PEDV strain SHpd/2012 [28]. However, DNA-PK phosphorylation on Ser2056 was not consistently affected by CV777 or HLJBY infection (Figure 1A,B). In contrast, UV-inactivated PEDV did not affect DDR-related proteins (Figure 1A), indicating that active virus replication is essential in order to activate cellular DDA. Similarly, we observed increased levels of phospho-ATM, phospho-Chk2, and γH2AX but not phospho-ATR in CV777-infected Marc145 cells (Figure 1C), demonstrating that PEDV infection-induced ATM signaling was not cell-type-specific. In addition, the proteolytic cleavage of PARP increased concomitantly with γH2AX at 36 hpi in both Vero-E6 and Marc145 cells (Figure 1A–C), implying that γH2AX probably correlates with the activation of caspases and apoptosis at the late stage of infection.

### 3.2. Inhibition of ATM or Chk2 Suppresses PEDV Early Replication

**Figure 2:**
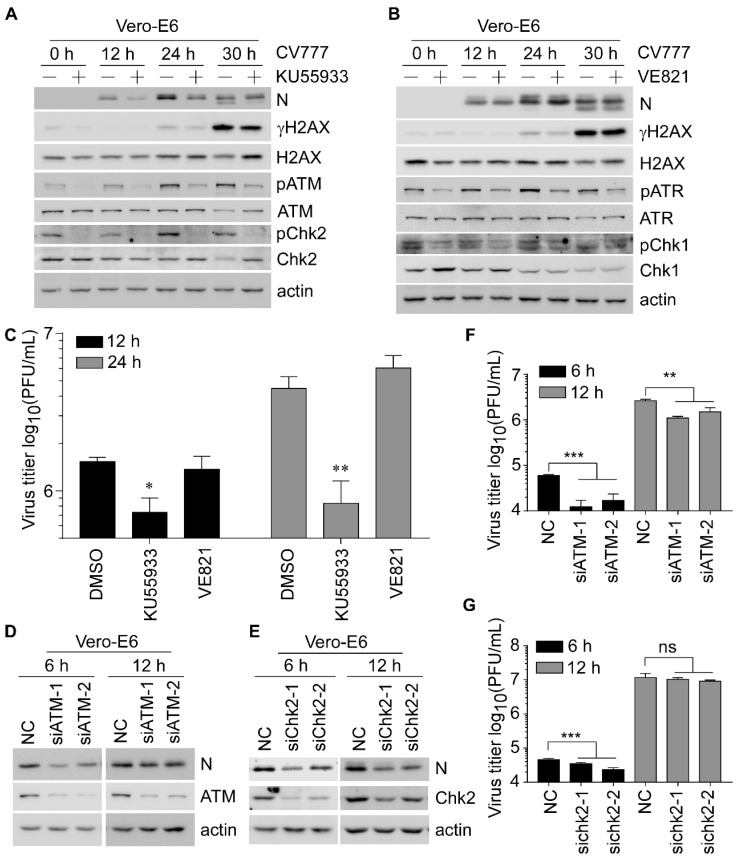
Suppression of the ATM signaling pathway inhibits the replication of PEDV. (**A**,**B**) Western blots were prepared with extracts from Vero-E6 cells pretreated with DMSO, ATM antagonist KU55933 (6 μM) (**A**), or ATR antagonist VE821 (2 μM) (**B**) for 2 h and followed by infection with CV777 (0.5 MOI) in the presence of DMSO or KU55933 (**A**), or VE821 (**B**) for 12, 24, 30 h. (**C**) The virus titers were measured by PFU assay with supernatants harvested from Vero-E6 cells pretreated with DMSO or KU55933 (6 μM) or VE821 (2 μM) for 2 h before and during infection with CV777 (0.1 MOI) for 12 and 24 h. (**D**,**E**) Western blots were prepared with extracts from Vero-E6 cells transfected with scramble or 50 nM siATM (**D**), or siChk2 (**E**) for 48 h and followed by infection with 0.1 MOI CV777 for 6 and 12 h. (**F**,**G**) The experiment was performed as in (**D**,**E**), except the cells and supernatants were collected for PFU assay (* *p* < 0.05; ** *p* < 0.01; *** *p* < 0.001).

Since both phospho-ATM and -ATR were activated in Vero-E6 cells upon PEDV infection, we examined the effect of KU55933 (ATM-specific inhibitor) and VE821 (ATR-specific inhibitor) on PEDV replication to determine the role of DDR kinases in regulating viral replication. First, the CCK-8 assay was performed in Vero-E6 cells treated with an increasing dose of KU55933 or VE821 and showed that cell viability was not affected by tested concentrations of both inhibitors (Figure S1A,B). Next, the levels of DDR and viral proteins were examined by Western blot. We found that PEDV-induced accumulation of phospho-ATM/ATR and -Chk2/Chk1 were dramatically attenuated by KU55933/VE821 treatment at 12 to 30 hpi (Figure 2A,B and Figure S1C,D). However, the expression levels of PEDV N were decreased at 12 to 30 hpi by KU55933 (Figure 2A and Figure S1C), but not VE821 treatment (Figure 2B and Figure S1D). PEDV *ORF3* gene is related to viral replication [29,30]. Thus, mRNA levels of *ORF3* and extracellular virus titers were examined. We showed that the levels of *ORF3* mRNA and PEDV titers were only decreased in KU55933-treated cells at 12 and 24 hpi (Figure S1E,F and Figure 2C). Meanwhile, we also noticed that this inhibitory effect of KU55933 gradually compromised along with increased infection time, indicating that the ATM kinase mainly contributes to PEDV replication at the early stage. To determine which factor of the ATM signaling pathway was involved in PEDV early replication, the protein level of PEDV N was measured in the presence or absence of ATM or Chk2. The results showed that knockdown of ATM/Chk2 led to reduced expression of PEDV N at 6 and 12 hpi (Figure 2D,E and Figure S2A,B). Consistently, the PEDV titers at 6 and 12 hpi were also decreased when ATM was knocked down (Figure 2F). However, the reduction of PEDV titers was less pronounced at 12 hpi than 6 hpi upon knockdown of ATM, suggesting ATM was involved in the early stage of viral life cycle. In addition, the PEDV titers were slightly decreased by Chk2 knockdown at 6 hpi, but not at 12 hpi (Figure 2G), indicating Chk2 as a downstream effector of ATM plays a minor role in viral replication. Collectively, these findings indicated that ATM might play a role in the early stage of PEDV replication.

### 3.3. The Level of Phospho-ATM Is Correlated with PEDV-Induced ROS

**Figure 3:**
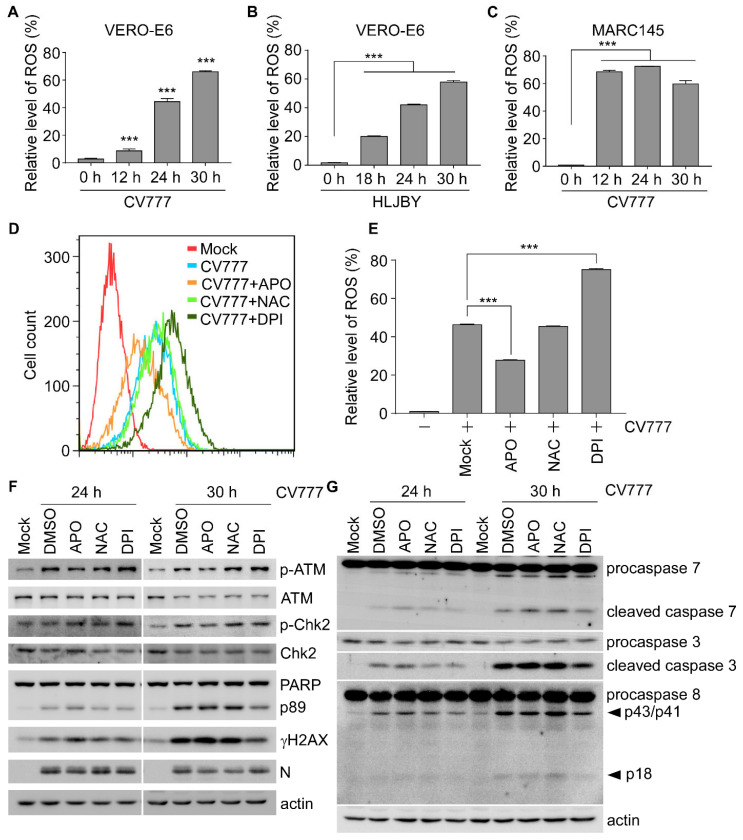
PEDV-induced cellular ROS contributes to the activation of the ATM signaling. (**A**) ROS levels were measured with Vero-E6 cells uninfected or infected with CV777 (1 MOI) for 12, 24, and 30 h. The relative ROS levels were calculated by the DCF fluorescence intensity in PEDV-infected cells. Results are representative of three independent experiments. (**B**) ROS levels were measured with Vero-E6 cells uninfected or infected with HLJBY (1 MOI) at 18, 24, and 30 h. (**C**) The experiment was performed as in A, except using Marc145 cells. (**D**) ROS levels were measured in Vero-E6 cells pre-treated with DMSO, APO (1 mM), NAC (100 μM), or DPI (0.5 μM) for 2 h and followed by CV777 (1 MOI) infection in the presence of DMSO, APO (1 mM), NAC (100 μM), or DPI (0.5 μM) for 24 h. (**E**) The quantification of the relative ROS levels calculated by the DCF fluorescence intensity in cells that were treated as in (**D**). Results are representative of three independent experiments. (**F**,**G**) Western blots were prepared with extracts from Vero-E6 cells treated with DMSO, APO (1 mM), NAC (100 μM), or DPI (0.5 μM) for 2 h before and during infection with CV777 (1 MOI) for 24 and 30 h (*** *p* < 0.001).

Previous reports showed that PEDV infection induces reactive oxygen species (ROS) production and leads to apoptosis and autophagy [31,32]. ROS are the byproduct of mitochondrial physiological activities and able to stimulate DNA damage. To explore the role of ROS in PEDV-induced DDR, we examined the cellular ROS level in PEDV-infected cells. The results revealed the robust activation of ROS production in Vero-E6 (Figure 3A,B) and Marc145 cells (Figure 3C) upon PEDV infection. Next, PEDV-induced ROS pro duction was analyzed by using three antioxidants, including apocynin (APO), an Nicotinamide adenine dinucleotide phosphate (NADPH) oxidase inhibitor, diphenyleneiodonium chloride (DPI), an inhibitor of NADPH oxidase and iNOS/eNOS, and N-acetylcysteine (NAC), a scavenger of free radicals especially oxygen radicals [33]. We showed that PEDV-induced accumulation of ROS was reduced by about 40% by APO treatment (46.3% in CV777 vs. 27.7% in APO+CV777), but was not affected by NAC treatment (Figure 3D,E). Surprisingly, the level of PEDV-induced ROS increased by around 62.2% in DPI-treated cells (46.3% in CV777 vs. 75.1% in DPI+CV777) (Figure 3D,E). Consistently, phospho-ATM exhibited the same trend as the cellular ROS level in control or PEDV-infected cells treatment with mock, APO, NAC, or DPI (Figure 3F). Surprisingly, we noticed that the levels of cleaved PARP and γH2AX were decreased concurrently in DPI-treated cells at 30 hpi regardless of increased phospho-ATM (Figure 3F), implying the existence of other factors in regulating H2AX phosphorylation. Given that the level of cleaved PARP and γH2AX changed simultaneously (Figure 1 and Figure 3F), we speculated that γH2AX was associated with the status of apoptosis. To address this, we examined the caspases’ cleavage upon antioxidants treatment, including executioner caspase-3 and -7 as well as the initiator caspase-8. As expected, the levels of cleaved caspase-3, -7, and -8 were decreased concomitantly with the reduced accumulation of γH2AX upon DPI treatment, but not APO or NAC treatment (Figure 3G).

### 3.4. PEDV Infection Induces a Confluent Pattern of γH2AX-Nuclear Staining

**Figure 4:**
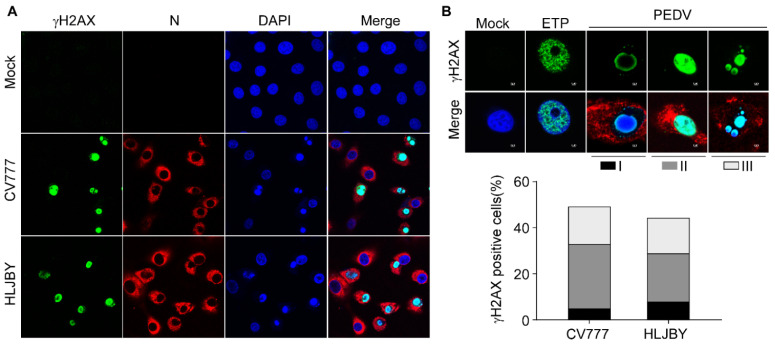
The pan-nuclear staining of γH2AX in PEDV-infected Vero-E6 cells. (**A**) Confocal images are representative of γH2AX immunofluorescence staining in Vero-E6 cells infected with PEDV (1 MOI) for 30 h. γH2AX was labeled in green, PEDV N was labeled in red, and nuclei were stained in blue with DAPI. (**B**) Representative confocal images of a single cell showing the γH2AX patterns in different phases (600× magnification). From left to right: untreated cells, etoposide-treated cells, and CV777 infected cells. The graph shows the relative distribution of the different γH2AX patterns (over 100 cells). Black columns correspond to peripheral nuclear staining (ring pattern, I), dark gray columns correspond to pan-staining (flooded pattern, II), and light gray columns correspond to apoptotic bodies fully stained with γH2AX (III).

Typical γH2AX foci by IFA have been widely used as a biomarker of DNA double-strand breaks (DSBs) upon treatment with genotoxic reagents or irradiation [34]. Therefore, we analyzed the nuclear immunostaining of γH2AX in PEDV-infected Vero-E6 cells (Figure 4A). Unlike etoposide, a topoisomerase II inhibitor, induced bright punctate γH2AX foci distributed throughout the nucleus, PEDV-induced γH2AX staining tended to be a confluent pattern that could be subdivided into three phases (Figure 4B, upper panel), including phase I (nuclear ring staining), phase II (pan-nuclear staining), and phase III (co-staining with shrunken apoptotic bodies), which highly resembled TNF-related apoptosis-inducing ligand (TRAIL)-induced γH2AX activation [35,36]. Quantification of γH2AX staining in each phase showed that over 40% were nuclear γH2AX staining; among these, around 80% of γH2AX positive cells were in phases II and III, consistent with the progression of cell death upon PEDV infection (Figure 4B, lower panel).

### 3.5. PEDV-Induced γH2AX Is Associated with Caspase Activation

**Figure 5:**
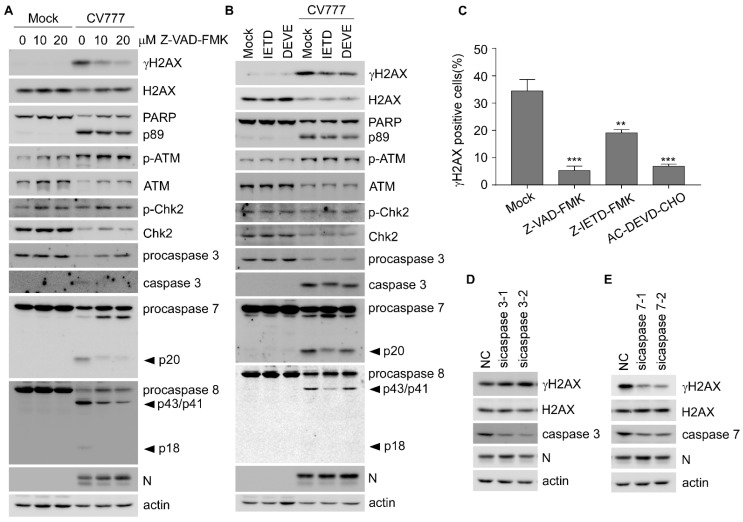
PEDV-induced phosphorylation of H2AX is decreased by caspase inhibitors. (**A**) Western blots were prepared with extracts from Vero-E6 cells treated with DMSO or caspase pan-inhibitor Z-VAD-FMK (0, 10, and 20 μM) for 2 h before and during infection with CV777 (0.5 MOI) for 30 h. (**B**) Western blots were prepared with extracts from Vero-E6 cells treated with DMSO or caspase-8 inhibitor Z-IETD-FMK (50 μM), or caspase-3/7 inhibitor Ac-DEVD-CHO (50 μM) for 2 h before and during infection with CV777 (0.5 MOI) for 30 h. (**C**) IFA was performed with Vero-E6 cells treated with DMSO, Z-VAD-FMK (10 μM), Z-IETD-FMK (50 μM), and Ac-DEVD-CHO (50 μM) for 2 h, respectively, before and during infection with CV777 (0.5 MOI) for 30 h. The cells were fixed and double-immunostained with specific rabbit anti-γH2AX and mouse anti-N antibodies. The nuclei were stained with DAPI. The graph shows the percentage of γH2AX positive cells in about 200 PEDV-infected cells. (**D**,**E**) Western blots were prepared with extracts from Vero-E6 cells transiently transfected with 50 nM scramble or caspase-3/7 siRNA for 48 h, followed by 1 MOI CV777 infection for 25 h (** *p* < 0.01; *** *p* < 0.001).

To determine the role of caspases in PEDV-induced H2AX phosphorylation, Vero-E6 cells were treated with the pan-caspase inhibitor Z-VAD-FMK, and we found that γH2AX was dramatically decreased along with reduced cleavage of caspase-3, -7, and -8, in Z-VAD-FMK-treated cells in a dose-dependent manner, while phosphor-ATM and -Chk2 were not affected (Figure 5A). Next, to determine which caspase is involved in regulating H2AX phosphorylation, Vero-E6 cells were treated with Z-IETD-FMK (a caspase-8 inhibitor) and Ac-DEVD-CHO (a caspase-3/7 inhibitor). We found that both inhibitors exerted similar inhibitory effects on γH2AX, but not phospho-ATM and -Chk2, suggesting that PEDV-induced γH2AX was associated with caspase activity but not phospho-ATM and -Chk2 (Figure 5B). However, the levels of cleaved caspase-3, -7, and -8 were decreased in Z-IETD-FMK-treated cells, while only caspase-3 and -7 were inhibited in Ac-DEVD-CHO-treated cells, indicating that caspase-3/-7 might contribute to the PEDV-induced H2AX phosphorylation (Figure 5B). Next, we also analyzed the γH2AX staining and found the percentage of γH2AX positive cells was significantly decreased by caspase inhibitors (Figure 5C and Figure S3). To further clarify the major caspase involved in this regulation, caspase-3 or -7 was transiently knocked down by siRNA in Vero-E6 cells. We showed that PEDV-induced γH2AX was only inhibited in caspase-7 knockdown cells (Figure 5D,E), suggesting that caspase-7 is a key factor that mediates PEDV-induced H2AX phosphorylation. Taken together, these data demonstrate that PEDV regulates H2AX phosphorylation through activating caspase-7 rather than the classical ATM-Chk2 signaling pathway.

### 3.6. Caspase-Activated DNAse Plays a Role in PEDV-Induced H2AX Phosphorylation

**Figure 6:**
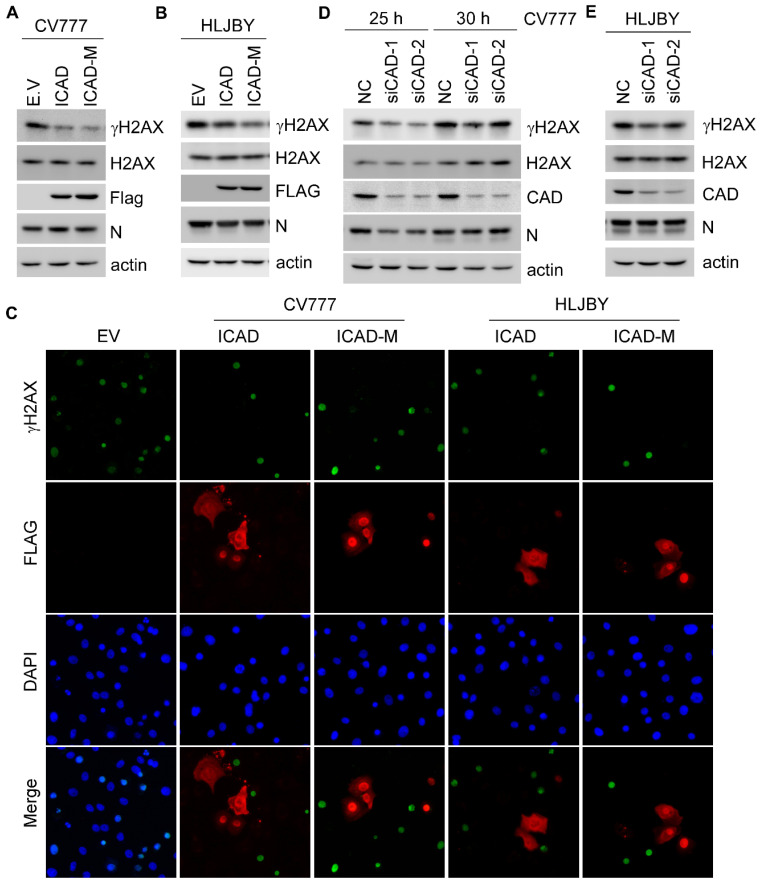
CAD implicates in PEDV-induced H2AX phosphorylation. (**A**,**B**) Western blots were prepared with extracts from Vero-E6 cells transiently transfected with empty vector or Flag-tagged ICAD or ICAD-M (D117E, D224E) for 30 h, followed by 1 MOI CV777 (**A**) or HLJBY (**B**) infection for 25 h. (**C**) IFA was performed with Vero-E6 cells transiently transfected with empty vector or Flag-tagged ICAD or ICAD-M (D117E, D224E) for 30 h, followed by 1 MOI CV777 infection for 25 h. (**D**,**E**) Western blots were prepared with extracts from Vero-E6 cells transiently transfected with 50 nM scramble or CAD siRNA for 48 h, followed by 1 MOI CV777 (**D**) or HLJBY (**E**) infection for 25 and 30 h.

Caspases-3 and -7 can cleave the inhibitor of caspase-activated DNAse (ICAD) and lead to dissociation of the caspase-activated DNAse (CAD) from the CAD-ICAD complex to form the CAD homodimer, which acts as a DNA scissor to generate DSBs and initiate apoptosis [37]. Studies showed that PEDV induces apoptosis at the late stage of infection [24,38] and increases γH2AX as a result of apoptotic DNA fragmentation [39]. To determine whether the activity of CAD is responsible for PEDV-induced γH2AX, Vero-E6 cells were transfected with Flag-tagged ICAD or ICAD-M (D117E, D224E) and followed by the infection of CV777 or HLJBY. We showed that PEDV-induced γH2AX was decreased in cells expressing ICAD and further decreased in cells expressing ICAD-M (Figure 6A,B), owing to the resistance of ICAD-M to caspase cleavage and form a stable inactive ICAD/CAD in cells. Moreover, γH2AX staining cells were also analyzed and showed that γH2AX was abolished in cells expressing ICAD or ICAD-M (Figure 6C). To further confirm this, endogenous CAD was transiently knocked down in Vero-E6 cells. The results showed that knockdown of CAD attenuated CV777- or HLJBY-induced H2AX phosphorylation (Figure 6D,E). Therefore, these data demonstrated that CAD activity is involved in PEDV-induced H2AX phosphorylation.

### 3.7. H2AX Contributes to the PEDV Early Replication

**Figure 7:**
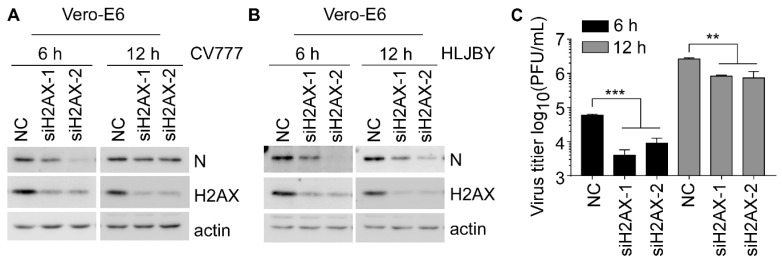
Silencing H2AX decreases PEDV replication. (**A**,**B**) Western blots were prepared with extracts from Vero-E6 cells transiently transfected with 50 nM scramble or H2AX siRNA for 48 h, followed by 1 MOI CV777 (**A**) or HLJBY (**B**) infection for 6 and 12 h. (**C**) Western blots were prepared with extracts from Vero-E6 cells transfected with 50 nM scramble or H2AX siRNA for 48 h, followed by 1 MOI CV777 infection for 6 and 12 h. The cells and supernatants were collected for PFU assay (** *p* < 0.01; *** *p* < 0.001).

The aforementioned studies showed that the PEDV-induced γH2AX occurred simultaneously with caspase-7 activation in an ATM-independent manner. Therefore, we investigated the role of H2AX in PEDV replication. We observed a reduced expression of PEDV N at 6 and 12 hpi in H2AX-knockdown cells (Figure 7A,B). Virus propagation measured by PFU also showed decreased PEDV titers upon H2AX knockdown (Figure 7C). These data together indicated that H2AX plays a role in regulating PEDV early propagation.

## 4. Discussion

**Figure 8:**
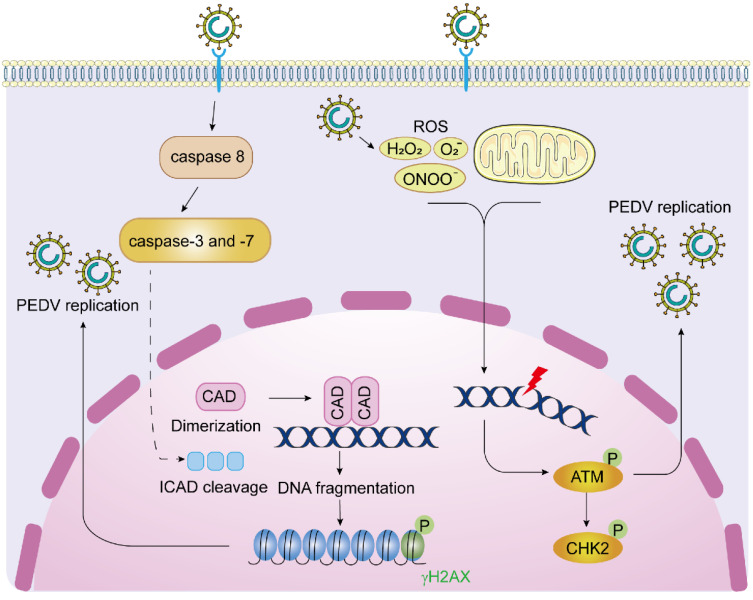
The interplay between PEDV replication and cellular DNA damage response. After PEDV enters host cells and replicates in the cytoplasm, the accumulation of massive intracellular ROS activates ATM-mediated DSB. Activated ATM and its downstream substrate Chk2 facilitate viral replication in the early infection stage. In the late stage of PEDV infection, the initiator caspase-8 activates the executioner caspase-3 and caspase-7 which subsequently cleave ICAD and release CAD from the ICAD-CAD complex and promote the homodimerization of CAD. The scissor-like CAD dimers create DSBs in the genome and DNA fragmentation, leading to H2AX phosphorylation in the nucleus. Inhibition of H2AX, ATM, and Chk2 dampens the replication of PEDV.

In this study, we described two distinct routes for PEDV to activate cellular DDR signaling molecules, phosphor-ATM and γH2AX, to promote viral replication (Figure 8). We observed robust activation of phosphor-ATM, phosphor-ATR, and phosphor-Chk2 at 24 to 48 hpi in Vero-E6 cells, while inhibition of ATM, but not ATR kinase activity dramatically reduces viral replication. Consistently, knockdown of ATM or Chk2 moderately decreases PEDV replication at the early stage, indicating that activation of the ATM signaling is favorable for PEDV replication. In contrast to DNA viruses, such as herpes simplex virus (HSV), which triggers rapid DDR shortly after infection [40,41], RNA viruses such as Newcastle disease virus (NDV) and Zika virus elicit DDR at the relative late infection stage [42,43]. NDV induces ATM-Chk2 axis by membrane fusion mediated syncytium formation [42], while Zika virus induces DDR by replicating in the endoplasmic reticulum (ER) where excessive cytoplasmic ROS is produced [44]. In addition, HCV has also been shown to induce DDR through ROS and NOS accumulation [6,7]. Here, we found that PEDV infection activates the ATM signaling at the late infection stage. Like Zika virus and HCV, PEDV exclusively replicates in cytoplasmic convoluted membranes. Consistently, we found that PEDV-induced ATM activation is closely related to the level of cellular ROS, providing a possible explanation as to how single positive-strand RNA viruses (replicate in the cytoplasm) induce ATM-mediated DNA damage response.

APO and DPI are NADPH oxidase inhibitors. NAC is a synthetic precursor of cysteine and glutathione and functions as a ROS scavenger. These inhibitors are applied in numerous studies to suppress oxidative stress, but the mechanisms of their activities are unclear. Surprisingly, our data suggested that only APO sufficiently decreased ROS, while NAC had little effect and DPI showed the opposite effect (Figure 3D,E). Consistently, there was evidence that DPI could exert the prooxidative functions in certain cell types and conditions [45,46,47]. DPI alters the redox metabolism by inhibiting the pentose phosphate pathway responsible for NADPH synthesis, thus making cells more prone to oxidative stress [46]. Therefore, the effects of inhibitors are largely cell-type and stimuli dependent; however, many studies neglected the importance of examining the actual ROS level upon inhibitors’ treatment. In addition, DPI has also been shown to have a paradoxical effect on inducing apoptosis. For example, DPI promotes mitochondrial superoxide-mediated apoptosis [48], while inhibiting the sodium deoxycholate-mediated apoptosis [49]. DPI suppresses *H. pylori*-induced apoptosis and DNA fragmentation in gastric epithelial cells [50]. Consistently, our findings also suggest that DPI dramatically inhibits PEDV-induced cleavage of caspase-3, -7, -8, and PARP (Figure 3G), indicating the cytoprotective effect of DPI in response to PEDV infection.

Phosphorylation of H2AX, a variant of histone H2A, on serine 139 (γH2AX) has been widely considered a DSB marker and is essential for DNA repair [51,52]. Aside from canonical functions, many researchers reported multiple non-canonical functions of H2AX, including senescence maintenance [53], chromatin regulation during mitosis [54], stem cell development [55,56], and apoptosis [39]. We found that PEDV induces robust H2AX phosphorylation concomitantly with PARP cleavage at 36 hpi, indicating the initiation of apoptosis (Figure 1). Interestingly, a unique γH2AX nuclear staining pattern was observed which started with the perinuclear ring and evolved into pan-nuclear staining before and after nuclei undergo pyknosis, which highly resembles the γH2AX staining in TRAIL-treated cells [34,35]. γH2AX pan-nuclear staining is observed under many circumstances, including UV radiation [57,58], replicative stress and checkpoint abrogation [59,60], hypotonic treatment [61], ion irradiation [62], and viral infection [8,63,64]. Interestingly, TRAIL-induced activation of ATM and Chk2 can be blocked by a pan-caspase inhibitor [35], whereas treatment of Z-VAD-FMK efficiently decreases γH2AX without affecting PEDV-induced phosphor-ATM and -Chk2, suggesting PEDV regulates H2AX phosphorylation independent of ATM activity. As the major executioner caspase, caspase-3 is considered to play a crucial role in regulating H2AX phosphorylation [65]. In addition, caspase-8 has also been shown to orchestrate H2AX phosphorylation [66]. Here, we demonstrate that activated caspase-7 is the key caspase in regulating H2AX phosphorylation upon PEDV infection. Next, we explored the connection between CAD activation and H2AX phosphorylation and for this regulation PEDV-induced H2AX phosphorylation was tightly related to CAD activation independent of ATM activity. Collectively, these findings extended our knowledge of the interaction between PEDV and host DNA damage response and shed light on the mechanisms of RNA viruses, especially coronaviruses, in regulating host DDR.

## Figures and Tables

**Table 1:** The sequences of primers were used in this study.

Name	Sequences
hICAD-EcoRF	5′-CCGGAATTCATGGAGGTGACCGGGGACGCCGGGG-3′
hICAD-XhoR	5′-CCGCTCGAGCTATGTGGGATCCTGTCTGGCTCGC-3′
ICAD-M1-F	5′-CACCCAGGATCCCCGGAATTCATGGAGGTGACCGGGGACG-3′
ICAD-M1-R	5′-TGCCCCGCTTTCTGTTTCATCTACATCAAAGGACTC-3′
ICAD-M2-F	5′-ATGAAACAGAAAGCGGGGCAGGGTTGAAG-3′
ICAD-M2-R	5′-TACCCGTTTCTACTGCATCCACCTCCTCACCA-3′
ICAD-M3-F	5′-GGATGCAGTAGAAACGGGTATCAGCAGAGAGACC-3′
ICAD-M3-R	5′-CCCTCTAGATGCATGCTCGAGCTATGTGGGATCCTGTCTGGCT-3′
ORF3-F	5′-TTTGCACTGTTTAAAGCGTCT-3′
ORF3-R	5′-AGTAAAAGCAGACTAAACAAAGCCT -3′
GAPDH-F	5′-AGGTCGGAGTCAACGGATTT-3′
GAPDH-R	5′-TAGTTGAGGTCAATGAAGGG-3′

**Table 2:** The sequences of siRNA were used in this study.

Name	Sequences
siCaspase 3-1	GGA CUG UGG UAU UGA GAC A
siCaspase 3-2	GAA GGU AGC AAC AGA AUU U
siCaspase 7-2	UCG AAA CGG AAC AGA CAA A
siATM-1	GCA GAA AUC UAU GCA GAU A
siATM-2	UGA UAG AGC UAC AGA ACG A
siChk2-1	GGA CUC AAG UGU CAC UGA A
siChk2-2	CCU CUC UCA UGA GAA CCU U
siH2AX-1	ACA AGA AGA CGC GAA UCAU
siH2AX-2	ACG ACG AGG AGC UCA ACA A

## Data Availability

Not applicable.

## Supplementary Materials

The following supporting information can be downloaded at: https://www.mdpi.com/article/10.3390/v14081782/s1, Figure S1: The treatment of KU559333, not VE821 decreases PEDV replication; Figure S2: Knockdown of ATM or Chk2 inhibits PEDV replication; Figure S3: The percentage of PEDV-induced γH2AX positive cells was decreased upon treatment with caspase inhibitors.

## Abbreviations

APO, Apocynin; ATM, Ataxia telangiectasia mutated; ATR, ATM and Rad3 related; CAD, Caspase-activated DNAse; Chk1, Checkpoint kinase 1; Chk2, Checkpoint kinase 2; DDR, DNA damage response; DNA-PK, DNA-dependent protein kinase; DPI, Diphenyleneiodonium chloride; DSB, DNA double-strand break; H2AX, Histone H2AX; HCV, Hepatitis Cvirus; HU, Hydroxyurea; IBV, Infectious bronchitis virus; NAC, N-Acetylcysteine; NADPH, Nicotinamide adenine dinucleotide phosphate; NO, Nitric oxide; NOS, Nitric oxide synthase; PARP, Poly (ADP-ribose) polymerase; PEDV, Porcine epidemic diarrhea virus; PFU, Plaque-forming unit; ROS, Reactive oxygen species; TRAIL, TNF-related apoptosis-inducing ligand.
