# Supplementary material for: Porcine Enteric Coronavirus PEDV Induces the ROS-ATM and Caspase7-CAD-γH2AX Signaling Pathways to Foster Its Replication
Source: Viruses. 2022 Aug 15;14(8):1782. doi: 10.3390/v14081782 (PMC9413700; doi:10.3390/v14081782)
Supplement: Supplementary file 1 [file viruses-14-01782-s001.zip › viruses-1864599-supplementary.pdf]

Supplementary Figure S1

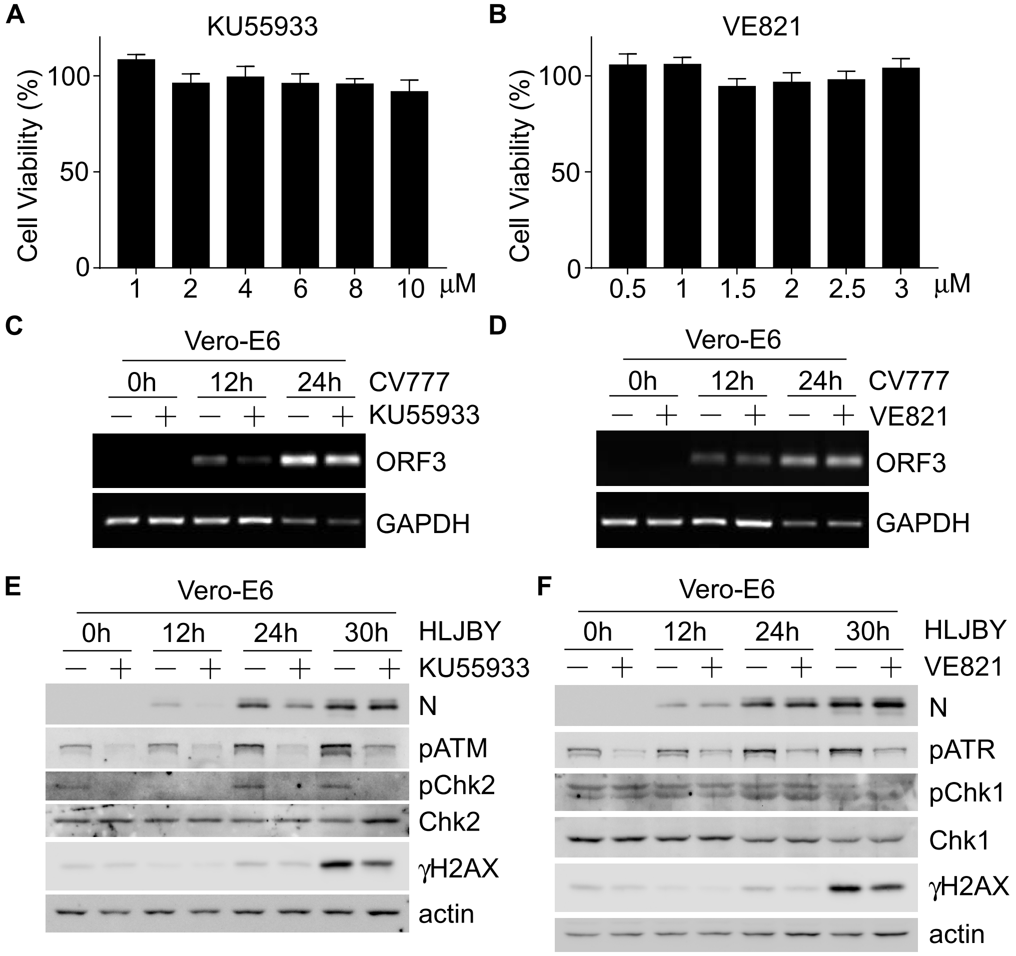

Figure S1. The treatment of KU559333, not VE821 decreases PEDV replication

(A) Vero-E6 cells were split into 96-well plates at 5000 per well. Cells were treated with KU55933(1, 2, 4, 6, 8, and 10 μM) for 24 hours and stained with CCK8 for 2 hours and the absorbance at 450 nM was measured. (B) The experiment was performed as in A, except the cells were treated with VE821 (0.5, 1, 1.5, 2, 2.5, and 3 μM). (C, D) RT-PCR was performed with RNA from Vero-E6 cells pretreated with DMSO, KU55933 (6 μM) (C) or VE821 (2 μM) (D) for 2 hours before and during infection with CV777 (0.5 MOI) for indicated time points. (E, F) Western blots were prepared with extracts from Vero-E6 cells pretreated with DMSO or ATM antagonist KU55933 (6 μM) (E) or VE821 (2 μM) (F) for 2 hours before and during infection with HLJBY (0.5 MOI) for indicated time points.

Supplementary Figure S2

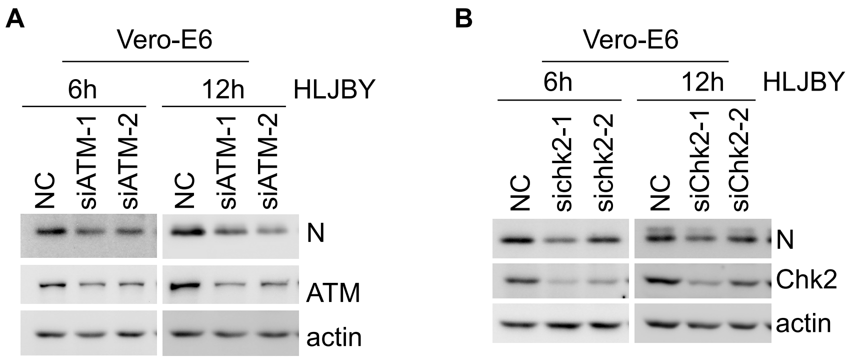

Figure S2. Knockdown of ATM or Chk2 inhibits PEDV replication
Western blots were prepared with extracts from Vero-E6 cells and were transfected with scramble or 50 nM siATM (A) or siChk2 (B) for 48 hours and followed by 0.1 MOI CV777 infection for indicated times.

Supplementary Figure S3

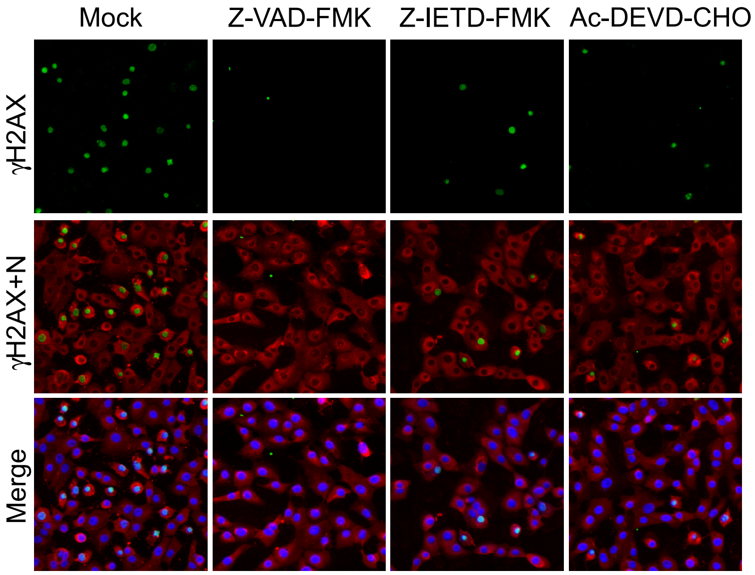

Figure S3. The percentage of PEDV-induced γH2AX positive cells was decreased upon treatment with caspases inhibitors

Vero-E6 cells were treated with DMSO or Z-VAD-FMK (10 μM) or Z-IETD-FMK (50 μM) or Ac-DEVD-CHO (50 μM) for 2 hours before and during infection with CV777(0.5 MOI) for 30 hours. The cells were fixed and double-immunostained with specific rabbit anti-γH2AX and mouse anti-N antibodies. The nuclei were stained with DAPI.
